# High Content Image Analysis of Cellular Responses of the Murine J774A.1 Cell Line and Primary Human Cells Alveolar Macrophages to an Extended Panel of Pharmaceutical Agents

**DOI:** 10.1007/s11095-024-03806-y

**Published:** 2025-01-07

**Authors:** Lysann Tietze, Laura Urbano, Stephan Eisenmann, Jacqueline Schwarzinger, Julia Kollan, Ben Forbes, Lea Ann Dailey, Gabriela Hädrich

**Affiliations:** 1 Department of Visceral, Transplant, Thoracic and Vascular Surgery, University of Leipzig Medical Center, 04103 Leipzig, Germany; 2 Department of Pulmonary Medicine, University Hospital of Halle-Wittenberg, 06120 Halle (Saale), Germany; 3 Department of Clinical, Pharmaceutical and Biological Sciences, University of Hertfordshire, College Lane, Hatfield, Hertfordshire AL10 9AB UK; 4 Institute of Pharmaceutical Science, Faculty of Life Sciences and Medicine, King’s College London, London, SE1 9NH UK; 5 Department of Pharmaceutical Sciences, University of Vienna, 1090 Vienna, Austria; 6 Vienna Doctoral School of Pharmaceutical, Nutritional and Sport Sciences, University of Vienna, 1090 Vienna, Austria

**Keywords:** cationic amphiphilic drugs, drug-induced phospholipidosis, high-content image analysis, human primary alveolar macrophages

## Abstract

**Introduction:**

*In vitro* screening of macrophages for drug-induced effects, such as phospholipidosis, is useful for detecting potentially problematic compounds in the preclinical development of oral inhaled products. High-content image analysis (HCIA) is a multi-parameter approach for cytotoxicity screening. This study provides new insights into HCIA-derived response patterns of murine J774A.1 cells and primary human alveolar macrophages (hAM).

**Methods:**

Several compounds were compared with reference groups (cationic amphiphilic drugs and apoptosis inducers) at different concentrations (0.01 to 10 µM). After incubation, cells were stained with fluorescence markers and HCIA was performed (Cytation™ 5 Cell Imaging System). Ten parameters were analysed: non-adherent cells, increased or reduced mitochondrial activity, membrane permeability, cell area, nuclear area, polynucleated cells, vacuole area, neutral and phospholipid content. A new system of response categorisation was developed for data analysis.

**Results:**

Murine J774A.1 cells exhibited a drug-induced response pattern that was distinct to the corresponding pattern of hAM cells. Comparison with the literature revealed that primary cells (rat or human origin) have similar response patterns, while cell lines (mouse, rat or human) exhibited a different response pattern. Hierarchical clustering revealed toxicologically aligned clusters of compounds, suggesting potential use for understanding mechanisms of drug effects in cell lines and primary cells.

**Conclusions:**

Valuable information for selecting a suitable cell type for HCIA screening of macrophage responses to drug compounds is provided. All cell types were suitable for screening drug-induced phospholipidosis. Still, human primary alveolar macrophages responded differently to drug treatment compared to macrophage cell lines and may be required to evaluate broader response-patterns and mechanisms of toxicity.

**Supplementary Information:**

The online version contains supplementary material available at 10.1007/s11095-024-03806-y.

## Introduction

The development of new oral inhaled drug products shows two important trends: 1) an increasing number of compounds with low solubility and 2) an increase in powder-based formulations for powder inhalers [1–3]. Currently, it is postulated that vacuolated macrophages may also be induced through phagocytosis of poorly soluble inhaled drug particles [4], which may lead to a temporary increase in vacuolated macrophages in the lung during drug treatment. When this effect is excessive, it is unclear whether these vacuolated macrophages contribute to pathological processes or constitute a non-adverse subclinical response to inhaled poorly soluble matter [5].

In 2014, Cook *et al*. reported that unacceptable safety was the main source of compound failures in development at AstraZeneca. The majority of preclinical safety failures could be attributed to specific organ toxicities [6]. As a result, most new compounds undergo an extensive *in vitro* screening process before progressing to non-clinical safety assessments [7, 8]. Murray *et al*. (2016) described how AstraZeneca carries out multiple cell-based high throughput screens to measure the toxicity of compounds, which includes a cell viability screen followed by orthogonal assays to test cell membrane integrity (LDH and CellTox™ Green from Promega), as well as a four-parameter apoptosis assay [9]. On top of this, advances in computational methods are making *in silico* screens more relevant indicating that they may represent a feasible option for augmenting the current screening paradigm [10].

As described above, screening processes for lung-specific toxicity increasingly incorporate both cytotoxicity endpoints as well as drug-induced phospholipidosis (DIPL) [4, 11]. DIPL is a cellular disorder resulting in the accumulation of 100–2400 nm inclusion bodies consisting of lamellar phospholipid aggregates with a concentric coiled appearance. Phospholipid-rich inclusion bodies appear in various cell types, in the lung, liver, and kidneys, following exposure to cationic, amphiphilic drugs (CADs) [8, 12, 13]. It is proposed that CADs, which accumulate in acidic lysosomes due to their weakly basic nature, can bind to phospholipids and induce the formation of non-digestible aggregated phospholipid structures [13]. It remains unclear whether the development of phospholipid-rich inclusion bodies is simply a morphologic feature of DIPL or has direct pathological consequences. DIPL is usually reversible with the disappearance of cellular inclusion bodies upon the termination of drug treatment. However, due to the unknown toxicological impacts and the high number of therapeutic compounds associated with DIPL, there is significant interest in research to understand better the mechanisms of induction and pathophysiological implications [2, 11, 13].

The usefulness of *in vitro* high content image analysis (HCIA) pre-screening of drug compounds for DIPL and other toxicity endpoints relies on the assumption that the *in vitro* results will reflect the *in vivo* effects of drug compounds both in animal and human studies [14]. To this end, several studies have compared the responses of different cell lines (rat, mouse, and human-derived) [4, 15, 16] with primary macrophages from rat bronchoalveolar lavage [14, 16]; first using amiodarone as a well-documented model CAD inducer of DIPL [14–16], then expanding to a larger panel of compounds [4]. One of the most salient observations reported by this compilation of studies was that the response of two commonly used macrophage cell lines, rat NR8383 and human U937, to amiodarone was very similar, while primary rat alveolar cells derived from a bronchoalveolar lavage responded differently [16]. Specifically, the two cell lines showed dose-dependent increases in mitochondrial activity and phospholipid content when exposed to amiodarone at concentrations from 0.1, 1 and 10 µM at both 24 and 48 h. The primary rat alveolar macrophages, in contrast, showed elevated membrane permeability and vacuole area at 24 h, but unexpectedly did not show the same increases in phospholipid staining at 24 h as the cell lines [16]. The data suggest that the rat primary cells were either less responsive to amiodarone or possess a different kinetic profile of DIPL development.

Analysis of the studies mentioned above provide an indication that primary cells may exhibit an inherently different response pattern to cell lines [4, 16]; however data on primary cells is still scarce. To broaden our understanding of this phenomenon, the current study assessed the effect of amiodarone and a panel further compounds on primary human alveolar macrophages (hAM,) as compared to the murine monocyte-derived macrophage-like J774A.1 cell line, shown previously in Hoffman *et al*. (2015) to develop a robust response to amiodarone treatment over a similar concentration range (0.03–40 µM) [15].

The hAM were obtained from bronchoalveolar lavage of hospitalised patients undergoing bronchoscopy for various clinical assessments, e.g. diagnosis of interstitial pneumonia, microbiological lavage in chronic obstructive pulmonary disease (COPD) exacerbation, or investigations of suspicious pulmonary nodules. The choice of using the murine J774A.1 cell-line was based on the ease of handling and low variability exhibited by this cell line compared to the previously investigated semi-adherent rat NR8383 cell line, which caused difficulties in high-content imaging due to loss of adherent cells during the staining procedure [16].

The extended panel of tested compounds investigated in this study is provided in Table I. Representatives of most classes of orally inhaled drug products on the market were included alongside two reference groups, CADs and compounds inducing apoptosis. Amiodarone (3 µM), served as an in-plate control compound for assay validation purposes [15]. The response of macrophages to this panel of compounds was assessed using HCIA, whereby ten attributes describing cell number, metabolic activity (increased or decreased), membrane integrity, nuclear area, cell area, vacuole area, number of polynucleated cells, intracellular neutral lipid and phospholipid content were assessed. Taken together, these attributes can provide a more holistic picture of drug-induced cytotoxicity compared to single assay endpoints alone [4, 16].

**Table I:**
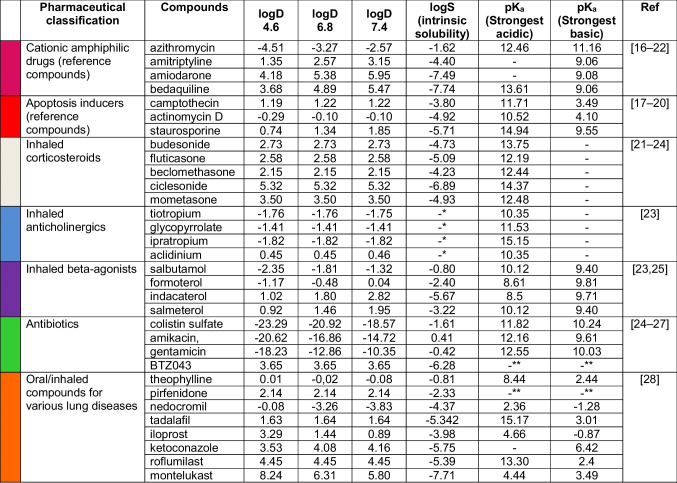
Test Compounds used for HCIA screening macrophage responses in J774A.1

Chemicalize was used for prediction of logD, logS and pKa properties (30.11.2024, http://chemicalize.com/, developed by ChemAxon)

* The molecule cannot be neutralized and the solubility prediction is not defined for molecules with non-zero charge

** pKa prediction cannot be performed since the molecule contains no ionizable atom forms

## Materials and Methods

### Materials

Foetal bovine serum (FBS Superior, Merck GmbH, Berlin, Germany), DMEM high glucose,1% penicillin/streptomycin (Gibco, Darmstadt, Germany), RPMI-1640, PBS, Hoechst 33342 MitoTracker® Image-IT™ DEAD Green™, HSC CellMask™ Deep Red HCS LipidTOX™ Red, HCS LipidTOX™ Green (Thermo), amiodarone, beclomethasone, µclear black 96-well plates (Greiner Bio-One, Frickenhausen, Germany), aclidinium, actinomycin D, amikacin, amitriptyline, bedaquiline, camptothecin, ciclesonide, colistin sulfate, gentamycin, glycopyrrolat, indacaterol, salbutamol, salmeterol, staurosporine (Biomol, Hamburg, Germany), azithromycin (LKT Laboratories, Inc, MN, USA), budesonide, formoterol, ipratropium, mometasone, rofluminast, tiotropium (TCI Deutschland GmbH,Eschborn, Germany).

#### Cells and Cell Culture

##### J774A.1

J77A.1 mouse monocyte-derived macrophage-like cells (ATCC®TIB-67™) were purchased from ATCC (Wesel, Germany) and used between passage number 8 and 30 from purchase. J77A.1 cells were seeded (2.6 × 10^4^ cells/cm^2^) onto flasks in culture medium DMEM high glucose completed with 10% foetal bovine serum and 1% penicillin/streptomycin. For subculturing, twice a week the cells were dislodged from the flask substrate with a cell scraper, aspirated and dispensed into new flasks in a ratio of 1:3. For experiments, 1.5 × 10^4^ cells were seeded onto bottom µclear black 96-well plates with the culture medium (100 µL/well). The cells were grown for 24 h until the treatment.

##### Human Alveolar Macrophages Obtained from Lavage (hAM)

Human alveolar macrophages were obtained from bronchoalveolar lavage from five individual donors. Bronchoscopy of hospitalised patients was performed for diagnostic purposes in cases of, for example, suspected interstitial pneumonia, microbial infections in chronic obstructive pulmonary disease (COPD) exacerbations or suspicious pulmonary nodules. Bronchoalveolar lavage (BAL) was obtained under an already Institutional Review Board (IRB) approved protocol (IRB Halle: 2012–055). BAL was performed according to a previously validated protocol [26]. Briefly, during flexible bronchoscopy (Olympus® BF190, Tokyo, Japan) the bronchoscope was placed in a wedge position in the previously determined segment. Using a dedicated catheter (Endoflex®, Voerde, Germany) through the working channel, saline at room temperature with a total volume of 100 – 200 mL was instilled in 3–5 aliquots and consecutively retrieved with low pressure. The first 2–3 portions were used for microbiological analysis, the final two aliquots for differential cytology (20 mL) and our current study (20 mL). The sample was immediately transferred to the lab for further processing. The suspension was filtered (100 µm filter) and centrifuged (400 g, 10 min) to obtain a cell pellet. Cells were resuspended in RPMI-1640 completed with 10% foetal bovine serum and 1% penicillin/streptomycin. For experiments, 1.5 × 10^4^ cells were seeded onto bottom µclear black 96-well plates containing complete RPMI-1640 medium without phenol red (100 µL/well). After 3 h cells were washed with PBS, removing all cell types except for adherent alveolar macrophages. Cells were then immediately incubated with cell culture medium (untreated group), compounds or controls for 48 h.

#### Treatment and Fluorescence Staining

J774A.1 cells were treated with 29 different compounds (*n* = 6, different passage numbers) at different subtoxic drug concentrations, depending on compound toxicity (0.01, 0.025, 0.05, 0.1, 1, 5 and 10 µM). hAM cells (*n* = 5 donors) were treated with only 5 and 10 µm concentrations of selected compounds from each group. J774A.1 cells were incubated for 48 h to provide a better comparison to Hoffman *et al*., 2023 [4] who show that morphological changes to amiodarone are more pronounced after 48 h of exposure. In contrast, hAMs showed a higher cytotoxicity at 48 h compound incubation and therefore were only incubated for 24 h with the compounds. Internal controls were included in each plate and consisted of 3 µM amiodarone (Sigma-Aldrich, Taufkirchen, Germany) to verify DIPL response. In each experiment, *n* = 6 wells per plate were used as untreated controls.

After incubation, cells were stained with different cocktails of up to four fluorescence markers to assess the ten attributes of cytotoxicity. The first dye cocktail consisted of 100 µL containing Hoechst 33,342 (10 mg/mL; nuclear dye), MitoTracker® (300 nM; mitochondrial activity) and Image-IT™ DEAD Green™ (35 nM; membrane integrity) for 30 min at 37°C. After a washing step with 100 µL PBS cells were fixed with 100 µL 4% paraformaldehyde for 15 min at room temperature in the dark. Fixed cells were stained overnight with HSC CellMask™ Deep Red (10 mg/mL; cytoplasm dye) after a second washing step. For the evaluation of lipid content, cells were incubated with HCS LipidTOX™ Red (phospholipid dye) diluted according to the manufacturer's protocol (1:1000). After washing with 100 µL PBS cells were fixed with Hoechst 33,342 (10 mg/mL; nuclear dye) in 4% paraformaldehyde for 30 min at room temperature in the dark. Following washing, the fixed cells were incubated with HCS LipidTOX™ Green (neutral lipid dye) diluted 1:1000 (according to the manufacturer's protocol) for additional 30 min at room temperature in the dark. Prior to imaging, cells were washed with PBS and plates stored at 4°C.

#### High Content Image Analysis

High content imaging was performed using the Cytation™ 5 Cell Imaging Multi-Mode Reader (BioTek, Bad Friedrichshall, Germany) with a 20-x objective in standard 2D imaging mode. Six pictures were acquired and analysed for each well using the Gen5 software (BioTek). The HSC CellMask™ Deep Red stain was used to identify single cells in each image for analysis of the fluorescence intensity of each marker on a single-cell basis. The Gen5 software was programmed to automatically calculate the percentage of abnormal cells in the treated cell population in each well compared to the mean values of the untreated cells in six wells on the same plate. As described previously [4, 15, 16], fluorescent markers were used to assess each attribute in untreated and treated cells. Cells with abnormal attributes were defined according to the criteria listed in Table II. The results from each analysis were then reported as a percentage of the cell population with abnormal attributes.

**Table II:** Cellular Attributes Measured to Assess Cytotoxicity and Definition of Abnormality Threshold Used in the Data Analysis

#	Attributes	Definition of the abnormality threshold
1	% non-adherent cells	(Mean cell number in well of untreated cells – mean cell number of wells with treated cells) / mean cell number in well of untreated cells) * 100
2	Elevated mitochondrial activity	Single-cell fluorescence intensity (FI) of the mitochondrial activity stain in the treatment group is > 2 SD of the mean FI of the untreated population
3	Reduced mitochondrial activity	Single-cell FI of the mitochondrial activity stain in the treatment group is < 2 SD of the mean FI of the untreated population
4	Elevated membrane permeability	Single-cell FI of the membrane integrity stain in the treatment group is > 2 SD of the mean FI of the untreated population
5	Abnormal nuclear area	Single-cell area of the nuclear stain in the treatment group is either > or < 2 SD of the mean nuclear area of the untreated population
6	Elevated cellular area	Single-cell area of the cytoplasm stain in the treatment group is either > 2 SD of the mean cellular area of the untreated population
7	Polynucleated cells	(Mean number of polynucleated cells in untreated cells –number of polynucleated cells in well of treated cells) / mean number of polynucleated cells in untreated cells) * 100
8	Elevated vacuole area	Single-cell area of the non-stained cytoplasm (vacuole space) in the treatment group > 2 SD of the area of the non-stained cytoplasm (vacuole space) of the untreated population
9	Elevated neutral lipids	Single-cell FI of the neutral lipid stain in the treatment group is > 2 SD of the mean FI of the untreated population
10	Elevated phospholipids	Single-cell FI of the phospholipid stain in the treatment group is > 2 SD of the mean FI of the untreated population

#### Statistical analysis

For hierarchical clustering, aggregated mean values for an increased percentage of cells with abnormal features were averaged and clustered for similarity, using hierarchical agglomerative clustering with Euclidean metric and Ward linkage, from the SciPy’s implementation of the algorithm and visualised as a cluster map. Discrete clusters were formed using the Agglomerative Clustering Scikit-learn implementation, setting the required number of clusters to 2, again with Euclidean metric and Ward linkage [27, 28].

## Results

### Attributes of Untreated J774A.1 Cells and hAM

**Fig. 1:**
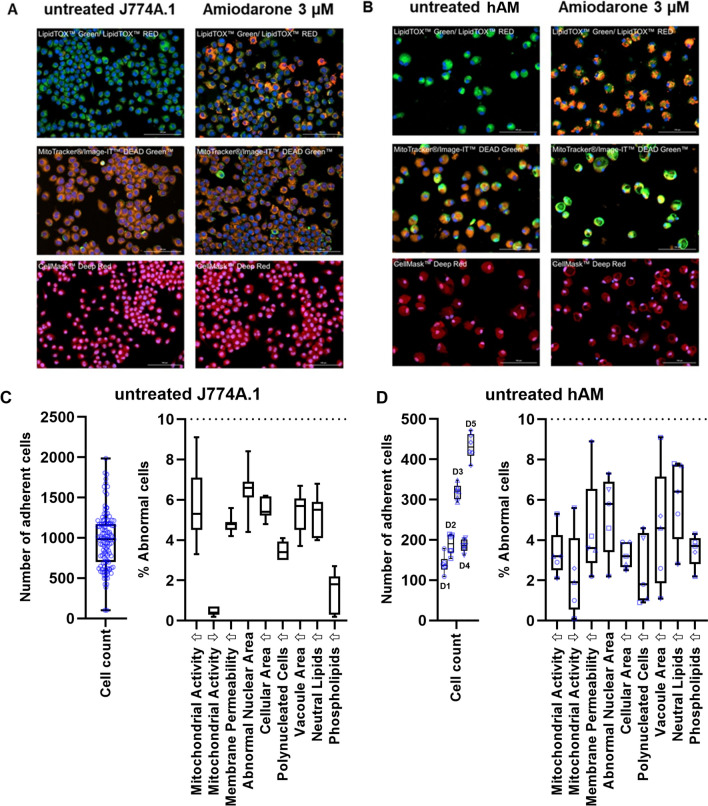
Images of fluorescently labelled (**A**) J774A.1 and (**B**) hAM comparing untreated (left panel) and amiodarone-treated (right panel) cells. Scale bar: 100 μm. The number of adherent cells (including box and whisker plots of quartiles, median, maximum and minimum values) are depicted for (**C**) untreated J774A.1 and (**D**) untreated hAM cells. The remaining nine HCIA parameters are shown in a separate diagram using box and whisker plots. Box limits depict the range of the central 50% of the data, with a line marking the median value. Whiskers show the minimum and maximum values. The data was calculated from *n* = 20 experiments (with six technical replicates each) for J774A.1 cells (passage numbers #4–30) and 5 different hAM donors (D1-5; with six technical replicates each).

The baseline metrics of the two different cell types were first established (Table III and Fig. 1). Due to species differences variations between primary cells and cell lines, and possible impacts of age and health status of the hAM donors, it was hypothesised that certain baseline attributes such as cell size and metabolic activity would differ and, secondly, that the hAM would show a higher variation due to interindividual differences in donors. Indeed, the mean cell area of the hAM was slightly higher (338 ± 53 µm^2^) than the J774.A1 cells (249 ± 58 µm^2^), however, the adherent hAM showed a narrower size range than the J774.A1 (252–393 µm^2^ *vs* 137–408 µm^2^, respectively). The hAM also showed a higher mitochondrial fluorescent staining intensity compared to J774.A1 and less variation in lipid staining intensity. The relative standard deviation (RSD%) for each attribute was comparable between the J774A.1 cell line and hAM primary cells. This was an important observation, as it indicated that the second standard deviation (2SD) as a threshold of cellular abnormality (Table II) would be comparable for both cell types. It also indicated that despite the expected interindividual variation in donors, the untreated hAM populations investigated in this study showed no obvious indications of pre-existing abnormality, at least concerning the attributes characterised here.

**Table III:** Attributes of Untreated J774A.1 Cells and hAMs Derived from Image Analysis. The Data was Calculated from *n* = 20 Experiments (with Six Technical Replicates Each) for J774A.1 Cells (Passage Numbers #8–30) and 5 Different hAM Donors (D1-5; with Six Technical Replicates Each)

Attribute	J774.A1					hAM				
	Mean	2SD	RSD%	Min	Max	Mean	2SD	RSD%	Min	Max
Cell number per well	1002	752	38	103	2138	260	224	43	109	472
FI (a.u.) MitoTracker®: mitochondrial activity	7126	4872	34	52	12876	14436	13920	48	7496	25765
FI (a.u.) Image-IT™ DEAD GreenTM: membrane integrity	5760	4252	37	3181	10637	6599	6424	49	2475	10104
Nuclear area (µm^2^)	101	38	19	75	135	94	16	9	84	105
Cell area (µm^2^)	249	116	23	137	408	338	106	16	252	393
Number of polynucleated cells / well	25	42	84	5	105	9	10	56	3	15
Vacuole area (µm^2^)	4	18	225	0	35	16	68	213	0	77
FI (a.u.) LipidTOX™ Green: neutral lipids	6453	6886	53	3404	16738	11492	5500	24	8645	15757
FI (a.u.) LipidTOX™ Red: phospholipids	2789	5148	92	0	9972	4343	6938	80	1448	10311

Following recommendations by Hoffman *et al*. (2023), a loss of > 50% of adherent cells compared to the untreated cells was defined as an exclusion criterium for HCIA [4]. Evaluation of the number of adherent cells following staining and washing indicated that even the untreated cell populations showed a high variation in cell number per well (Fig. 1C and D). This is due to the variable degree of cellular attachment to the culture plates and losses experienced during the extensive staining and washing procedures. Box and whisker plots depicting the median and the central quartiles indicate that the number of adherent J774A.1 cells was roughly symmetrical with the median value just slightly lower than the mean (Fig. 1C), which could indicate a nearly Gaussian distribution. The number of adherent hAM cells appeared to be donor-specific, although no donor-related trends were apparent for nine HCIA parameters (Fig. 1D).

The box and whisker plots of the percentage abnormal cells within the untreated cell populations highlighted that most data sets were unevenly distributed and therefore not likely to follow a Gaussian function (Fig. 1C, D). As indicated by the whiskers (maximum and minimum values), none of the untreated control groups showed more than 10% abnormal cells for any of the nine parameters evaluated. Similarly, Hoffman *et al*. (2023) proposed using the value of > 10% abnormal cells as a general threshold for treatment-induced effects. Their data was based on a similar, but not identical, HCIA protocol (i.e. different imaging instrument and software) which was used to characterise treated and untreated populations of rat NR8383 and human differentiated U937 cells. When combined, data from the two studies provides a strong indication that the 10% abnormality threshold may be applied across a wider range of cell types and species.

### Analysis of Data Variability in Treated Cell Populations Using Six Representative Compounds

**Fig. 2:**
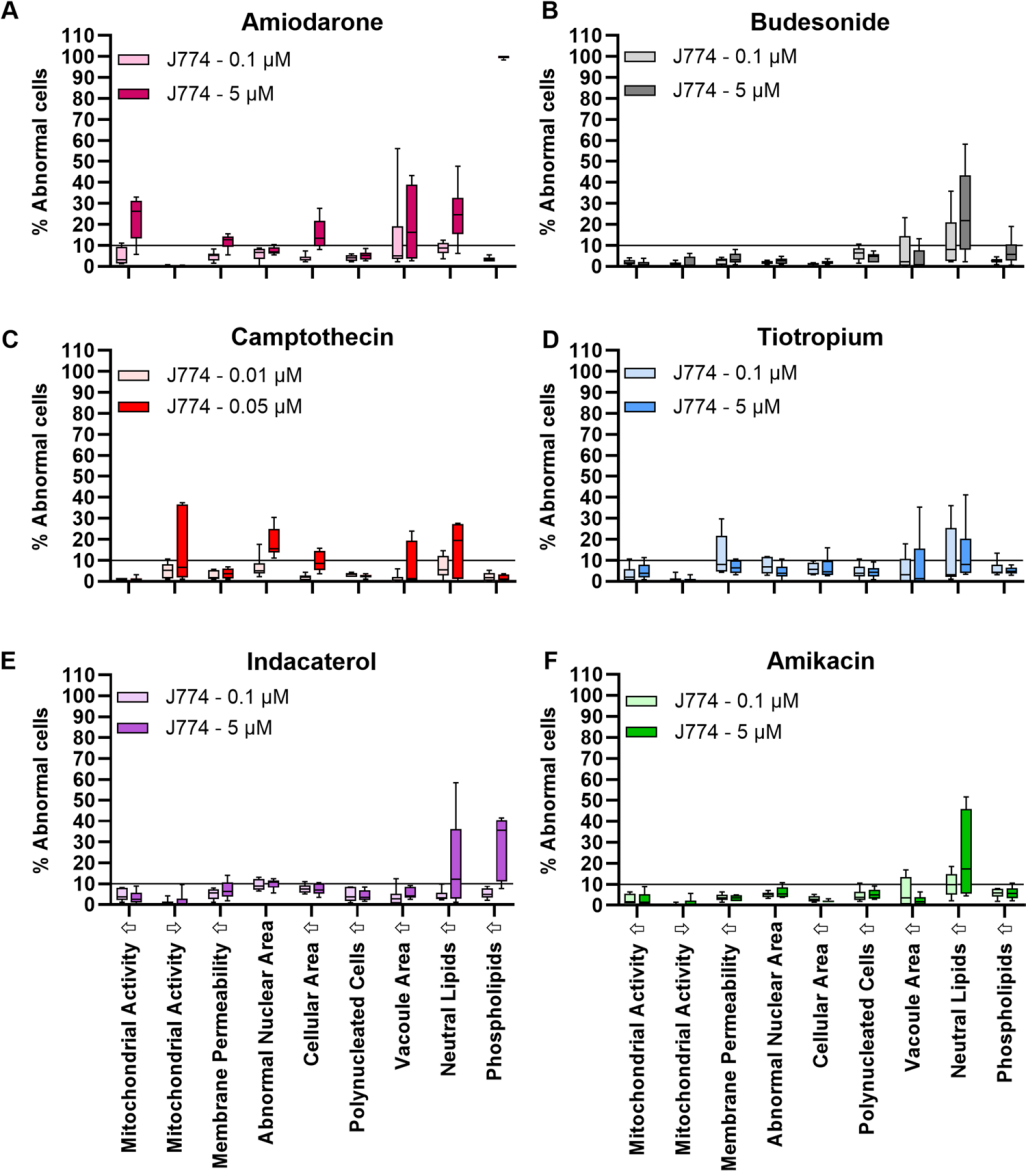
Data distribution (box and whisker plots) of the percentage of abnormal J774A.1 cells following 48 h incubation of a low and high dose of (**A**) amiodarone, (**B**) budesonide, (**C**) campothecin, (**D**) tiotropium, (**E**) indacaterol and (**F**) amikacin. Box limits depict the range of the central 50% of the data, with a line marking the median value. Whiskers show the minimum and maximum values from *n* = 6 experiments with different passage numbers. Note: Treatment of J774 with 5 µM amiodarone resulted in 98.3–100% elevated phospholipid staining. Due to the narrow distribution of the data, the box and whiskers in graph (**A**) are small but present in the top right corner.

To obtain detailed information on how drug treatment with subtoxic concentrations affects the data variability in both J774A.1 and hAM cells, six representative compounds (one from each pharmaceutical class) were chosen from Table I for analysis. J774A.1 cells were incubated for 48 h with both a low (0.1 µM) and high (5 µM) dose of each respective compound (Fig. 2). The apoptosis inducer, campothecin, which was toxic at these concentrations required lower doses (0.01 and 0.05 µM). In this data set, the treatment-related cell detachment did not exceed 50% (Table S1), indicating sufficient cells were available for robust HCIA analysis. The distribution data are shown in Fig. 2 as box and whisker plots for each parameter tested. Mean values, standard deviation and RSD% values are provided in Table S1 in the supplementary information for comparison.

The box and whiskers plots in Fig. 2 provide several insights, which were not as readily accessible in graphs depicting the individual data points or mean ± standard deviation values. First, it is easy to identify data sets where both the box and whiskers are visibly lower than the 10% threshold (roughly 33% of all data sets). In contrast, only one out of the 54 data sets has a distribution in which all six replicate values exceed the 10% threshold (i.e., amiodarone 5 µM; percentage of cells with elevated phospholipid content: 98.3–100%). The remaining 67% of data sets contain values which are both above and below 10% threshold, prompting the question of whether these outcomes should be classified as treatment-related or not.

Since this question was not addressed in previous studies [4, 16], we propose a revised system for categorising HCIA endpoints (Table IV). The revised system is simple and easy to apply to data sets with different replicate numbers or distribution functions. It also avoids the complexity of parametric or non-parametric statistical analyses, which are able identify significant increases in abnormal cell populations between the untreated *vs* treated groups but do not shed light on whether these differences are biologically or toxicologically meaningful. For example, applications of statistical tests to this data set (e.g., comparisons of % abnormal cells in treated and non-treated populations for all nine parameters) yielded several examples of statistically significant increases in the % of abnormal cells in the treatment groups, but often these increases were so minor that they were not biologically relevant (data not shown). Further, statistically significant differences were more likely to occur for sample sets with lower variability. As Fig. 2 clearly shows, low variability tends to correlate with negligible treatment-related effects, while parameters visibly altered by the treatment showed both a higher % of abnormal cells and a greater variability.

**Table IV:** Proposed Specifications for a Revised System to Categorise HCIA Endpoints

Category	Specification	Interpretation	Score
Normal	Median (Q2) < 10%	50% or more of the sample set is below the 10% threshold. The percentage of abnormal cells is similar to non-treated cell populations	1
Possibly treatment-related abnormalities	Q1 < 10% but median (Q2) > 10%	More than 50%, but not more than 75%, of the sample set exceeds the 10% threshold. Further replicate experiments are recommended to clarify the response	2
Likely treatment-related abnormalities	Q1 > 10%	More than 75% of the sample set exceeds the 10% threshold. The majority of the data set show treatment-related shifts in the population of abnormal cells	3
Treatment-related abnormalities	Minimum value > 10%	100% of the sample set exceeds the 10% threshold. The data set shows clear evidence for treatment-related shifts in the population of abnormal cells	4

### Analysis of Compound Treatment Across a Wide Dose Range in J774A.1 Cells

**Fig. 3:**
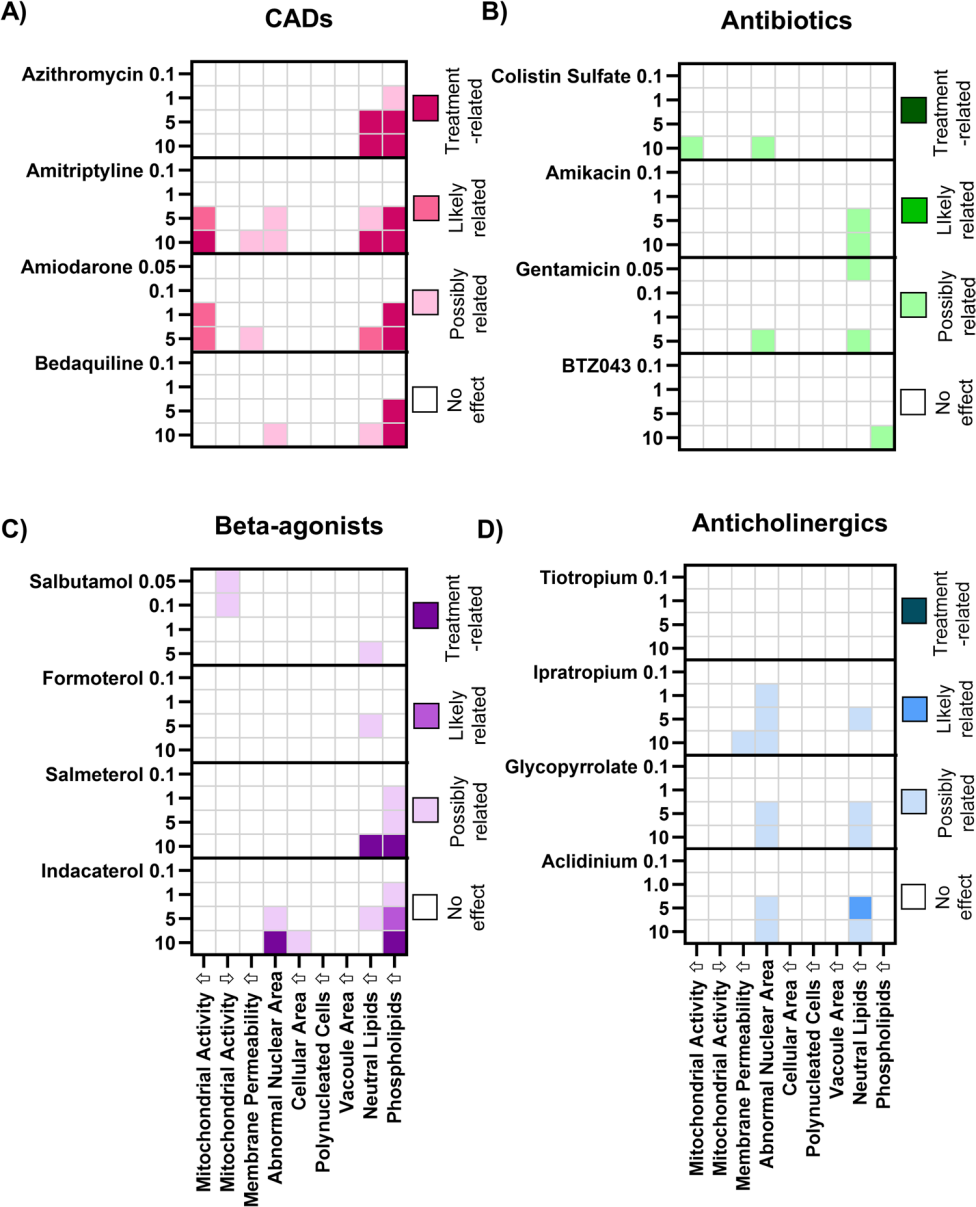
Categorisation of dose-dependent responses in J774A.1 cell populations incubated 48 h with increasing concentrations of test compounds grouped according to pharmaceutical class: (**A**) CADs, (**B**) antibiotics, (**C**) beta-agonists, and (**D**) anticholinergics. Categorisation values were derived from the data distribution of percentage of abnormal cells in J774.A1 cell populations from *n* = 6 experiments with different passage numbers. The numbers on the left y-axis denote the compound dose in µM. Compounds are listed in order of increasing lipophilicity (based roughly on log D) within the pharmaceutical classes. Individual values for all data points are listed in the supplementary information.

**Fig. 4:**
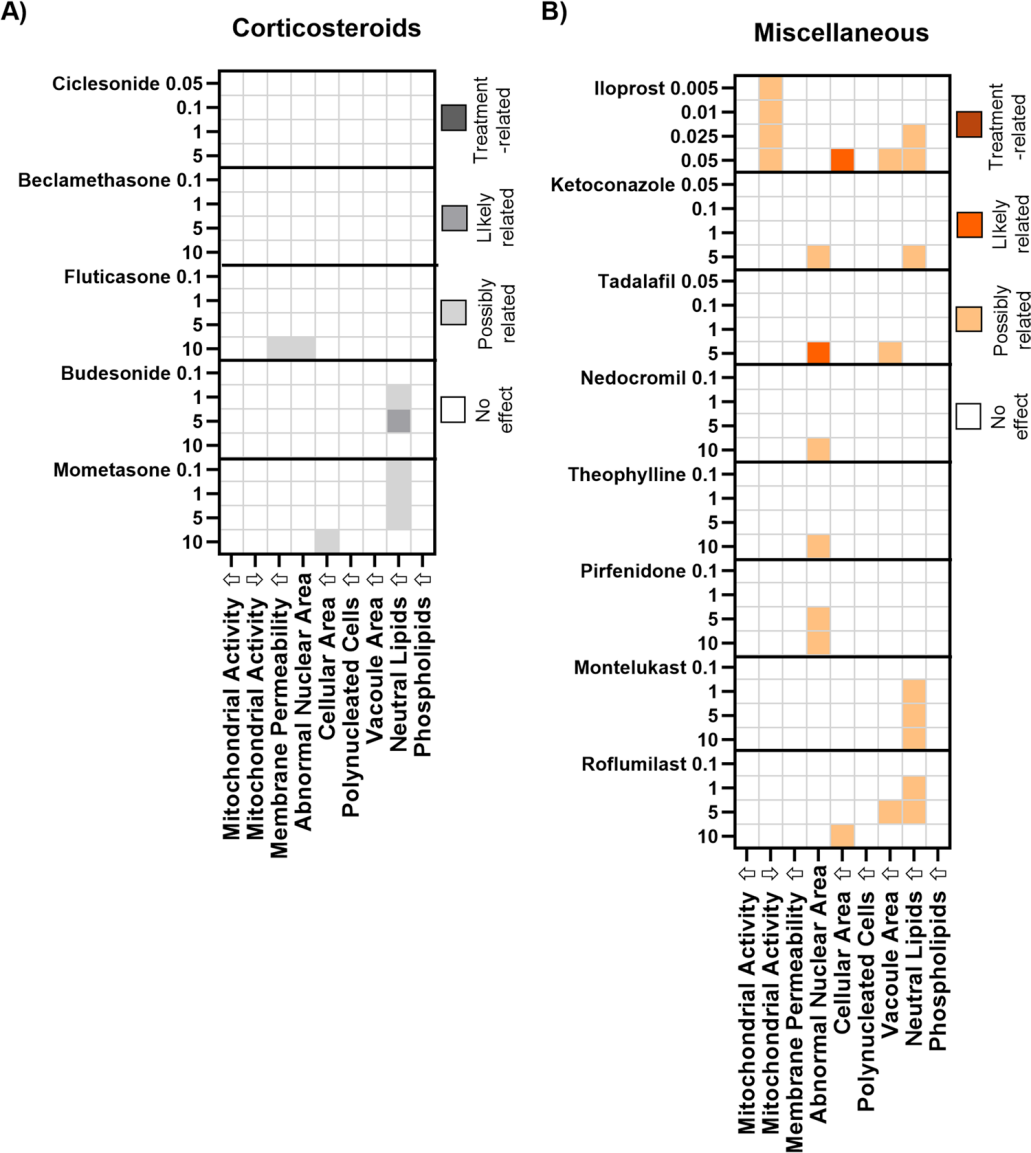
Categorisation of dose-dependent responses in J774A.1 cell populations incubated 48 h with increasing concentrations of test compounds grouped according to pharmaceutical class: (**A**) apoptosis inducers, (**B**) corticosteroids, (**C**) miscellaneous pharmaceutical classes. Categorisation values were derived from the data distribution of percentage of abnormal cells in J774.A1 cell populations from *n* = 6 experiments with different passage numbers. The numbers on the left y-axis denote the compound dose in µM. Compounds are listed in order of increasing lipophilicity (based roughly on log D values) within the pharmaceutical classes. Individual values for all data points are listed in the supplementary information.

The proposed categorisation scheme was then applied to a large data set containing the compounds listed in Table I. Compounds were incubated for 48 h with J774A.1 cells at increasing concentrations (Figs. 3 and 4) and data sets with > 50% cell adherence were included in the analysis. Due to excessive cell detachment even at the lowest dose tested (0.01 µM), staurosporine and actomycin D (apoptosis inducers) had to be excluded and are not shown. This left 29 remaining compounds which fulfilled the inclusion criteria for HCIA.

Heatmaps were used to depict treatment-related effects on the J774A.1 cells. Two main patterns can be identified. The first pattern was observed primarily in the CAD treatment group where the proportion of cells with highly elevated phospholipid levels exceeded the 10% threshold at the higher dose levels and in some cases (amiodarone and amitriptyline), the percentage of cells with elevated mitochondrial activity also exceeded 10%. The same response pattern was also observed in rat NR8383 and human U937 cells [4], but not in primary rat alveolar macrophages [16]. A similar observation was reported by Hoffman *et al*. (2015) for salbutamol [15], albeit at much higher drug concentrations. It was also noted that indacaterol and salmeterol also exhibited a treatment-related increase in cell population with elevated phospholipid staining intensity, corroborating a recent classification of both long acting beta-agonists as CADs due to their physicochemical properties (logP > 3;pKa > 6) [25].

The second response pattern identified in J774A.1 cells consisted of increases in the percentage of the population with both an abnormal nuclear area and elevated neutral lipid content. This response pattern was observed, for example, in cells treated with two out of four CADs and three out of four of the selected anticholinergics (Fig. 3A, D). An increased fraction of cells with elevated neutral lipids but no nuclear abnormalities was also observed in many treatment groups (Figs. 3 and 4), although this response was often categorised as “possibly” related to treatment rather than likely or definitively treatment-related.

Accumulation of neutral lipids in macrophage cells has recently been reported as a salient metabolic feature of phagocyte activation during infection and sterile inflammation [29] but has also been associated with stress in the endoplasmatic reticulum and autophagic processes [30]. Öhlinger *et al*. (2020) used HCIA to investigate neutral lipid accumulation in five macrophage models with different putative polarization states: 1) murine J774A.1 (cell line: resting state M0), 2) murine RAW264.7 (cell line: pro-inflammatory phenotype M1), 3) human THP-1 (cell line; differentiated with phorbol 12-myristate 13-acetate; polarisation state not described), and 4) human monocyte-derived macrophages (MDM) obtained from human peripheral blood monocytic cells and polarised via treatment to either the pro-inflammatory M1 phenotype or proliferative M2 phenotype. According to the literature, the pro-inflammatory M1 phenotype would be hypothesised to exhibit a higher neutral lipid accumulation following stimulus. Contrary to expectations, neutral lipid staining intensity was not significantly increased following exposure to amiodarone and chloroquine in the human derived M1 macrophages, but elevated levels were observed in the cell lines. The authors postulated that human primary cells may not be as responsive to stimuli as cell lines [31].

A closer look at the heatmaps (Figs. 3 and 4) shows that increases in abnormal nuclear area and neutral are mostly dose-dependent, but not in every case. This is attributable to differences in data distribution within the different dosing groups. In some cases, data at a lower dose may be more narrowly distributed and are therefore categorised as “likely treatment related”, while data from a higher dose may spread across a broader range, in which case the category “possibly” treatment related” is applied. This occurrence is shown in Figs. S1 and S2 of the supplementary information, where the two endpoints, abnormal nuclear area and elevated neutral lipid content, are depicted following treatment with increasing doses of beta-agonists (Fig. S1) and anticholinergics (Fig. S2). The box and whisker plots depicted in these figures clearly visualise how the spread of the data may impact the categorisation of the results. For full transparency, the supplementary information section also contains tables listing all replicate values, the calculated quartiles for each endpoint tested and categorisation scores. The raw data illustrates that there are some cases where the compound effects are very close to the 10% threshold (e.g. the lower quantile value for formoterol-induced elevation in neutral lipids was 9.9%). This again highlights that the classification system used is highly dependent on the data spread. An increase in replicates can be useful in such borderline cases.

Physical chemical properties (predicted solubility and partition coefficients) were also provided to explore whether compound properties correlate with the response patterns. No correlations were determined between *in vitro* response patterns and compound physicochemical properties. We therefore hypothesise that the response pattern indicates a general form of cell stress with an underlying mechanism that is more complex than compound lipophilicity alone. An expanded panel of endpoints (e.g. apoptosis markers etc.) would be needed to gain more mechanistic information.

### Effects of Compound-Treatment on hAM Cell Populations

**Fig. 5:**
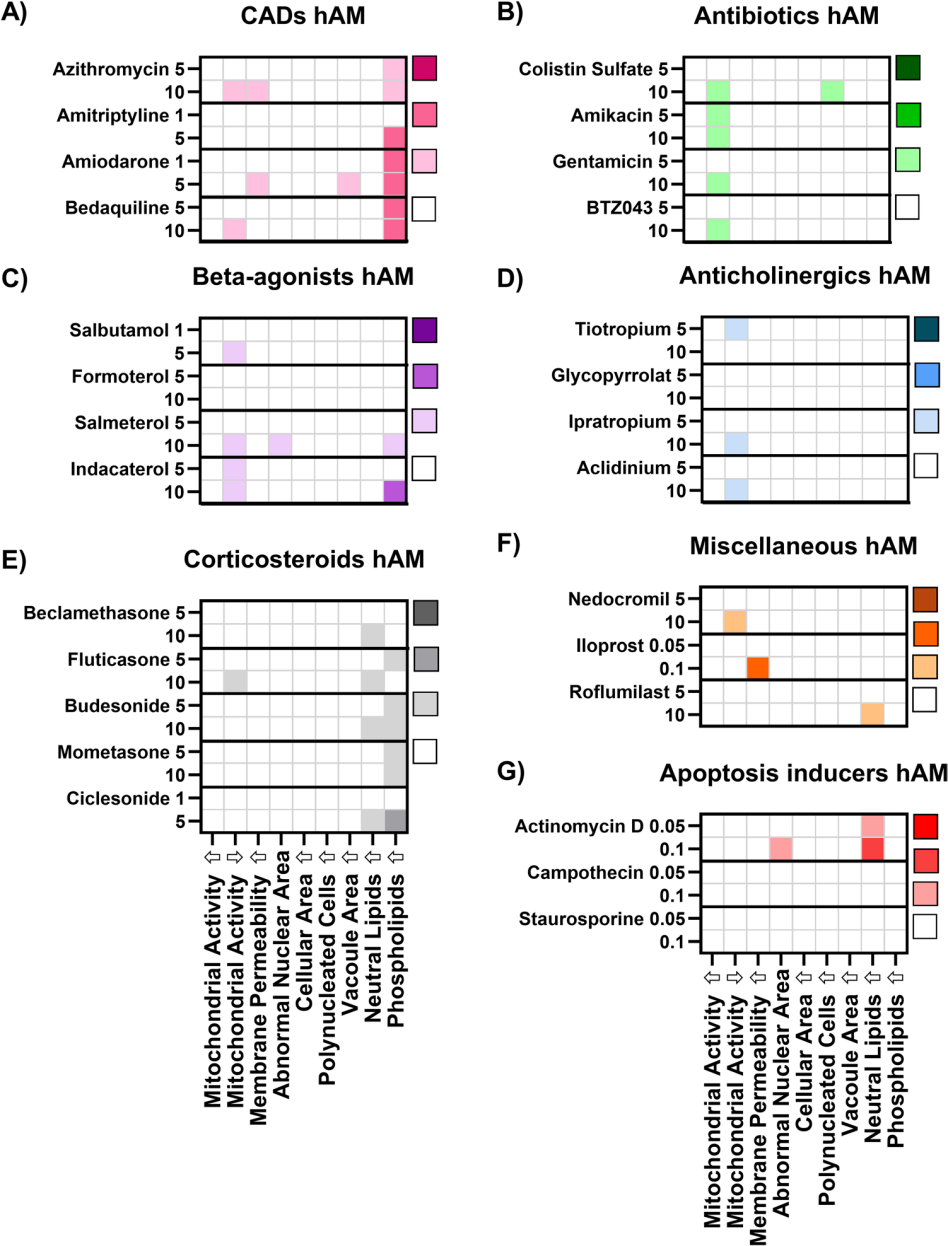
Influence of (**A**) CADs, (**B**) apoptosis inducers and (**C**) beta-agonists on the mean percentage of abnormal cells in hAM cell populations (*n* = 5 hAM donors). The numbers on the left y-axis denote the compound dose in µM. Compounds are listed in order of increasing lipophilicity (based roughly on log D values) within the pharmaceutical classes. Individual values for all data points are listed in the supplementary information.

The incubation of primary hAM with the same panel of pharmaceutical compounds (Fig. 5) showed a very different response pattern compared to J774A.1 cells. Incubation with CADs (24 h) was characterised by treatment-related increases in the percentage of cells with 1) reduced membrane integrity, 2) increased vacuole area per cell and 3) elevated phospholipid content (Fig. 5A). Interestingly, this response pattern closely resembles that of primary rat alveolar macrophages (also collected by lavage; [16]). Thus, the emerging picture from the growing body of literature on HCIA analysis of macrophage responses using different cell lines and primary cells [4, 16] is that primary cells show *response patterns* distinct from cell lines and the species of origin may be less important. However, it is equally valid to conclude that all cells tested in the current study and the literature show robust evidence of phospholipid accumulation following incubation with CADs. Therefore, if the primary research question is to determine whether a compound has the potential to induce DIPL (i.e. a single parameter), rapid screening of phospholipid staining in a representative cell line, such as the robust, easy-to-use J774A.1 cell line, will likely be the most efficient and predictive tool to answer this question. In such cases, the generation of a large HCIA data set may be more time-consuming without providing significant additional knowledge gain.

The second distinct pattern observed in the heatmap analysis of compound responses in hAM cells was prevalence of data sets associated with a higher percentage of cells with reduced mitochondrial activity (Fig. 5B-D). This parameter (i.e., reduced mitochondrial activity) was not included in earlier related studies [4, 16] so comparison data in primary rat alveolar macrophages is not available. Nearly all compound classes showed slight increases in the cell population with reduced mitochondrial activity. This was surprising because it was not observed at all in the J744 cell line. Secondly, it was also unexpected to see that treatment with the apoptosis inducers did not show this pattern, since this compound group should be associated with this effect. We hypothesise that the doses used in this group were not sufficient to cause a profound reduction in mitochondrial activity (> 2SD) in a majority of the cells, but further studies with higher doses would be required to confirm this.

Öhlinger *et al*. (2020) did compare the mitochondrial activity of primary human MDMs (M1 and M2 phenotypes) with that of macrophage-like cell lines following drug incubation and did not observe differences [31]. However, a CellTiter 96® Aqueous Non-Radioactive Cell Proliferation Assay was used, which measures dehydrogenase activity present in metabolically active cells [32]. In contrast, the MitoTracker® staining method used in the present study, as well as in Hofmann *et al*. (2017, 2023) [4, 16], is based on a cell-permeable probe with a thiol-reactive chloromethyl moiety, which passively diffuses across the plasma membrane and accumulates in active mitochondria [33]. Thus, differences in the underlying assay mechanism, as well as the data analysis may account for the different observations.

### Hierarchical Clustering Analysis

**Fig. 6:**
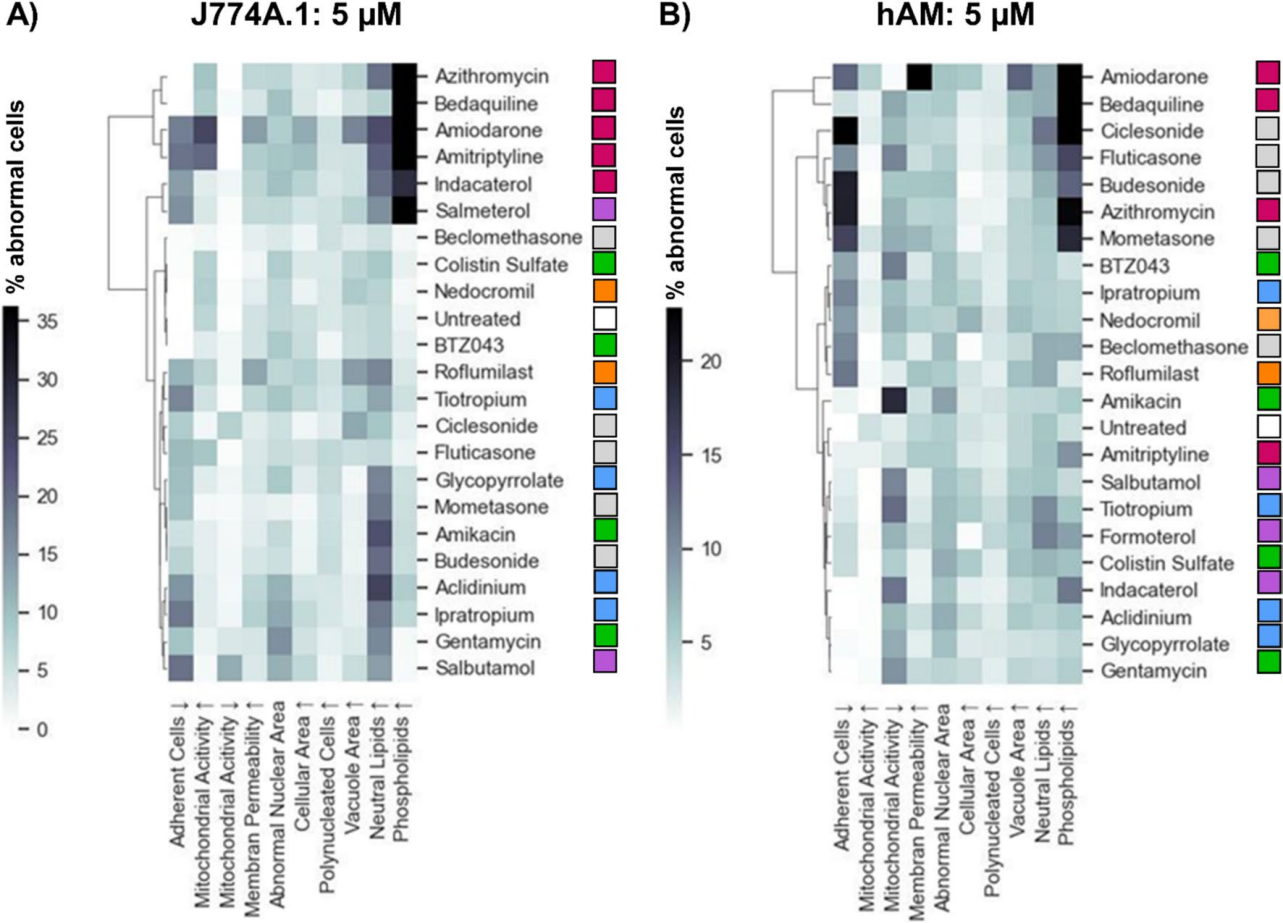
Hierarchical cluster map of (**A**) J774A.1 cells and (**B**) hAMs treated with selected compounds (5 µM). The heatmap values represent the mean increase in the percentage of abnormal cells. The color-coded squares next to each compound name indicate the pharmaceutical class (Table I).

**Fig. 7:**
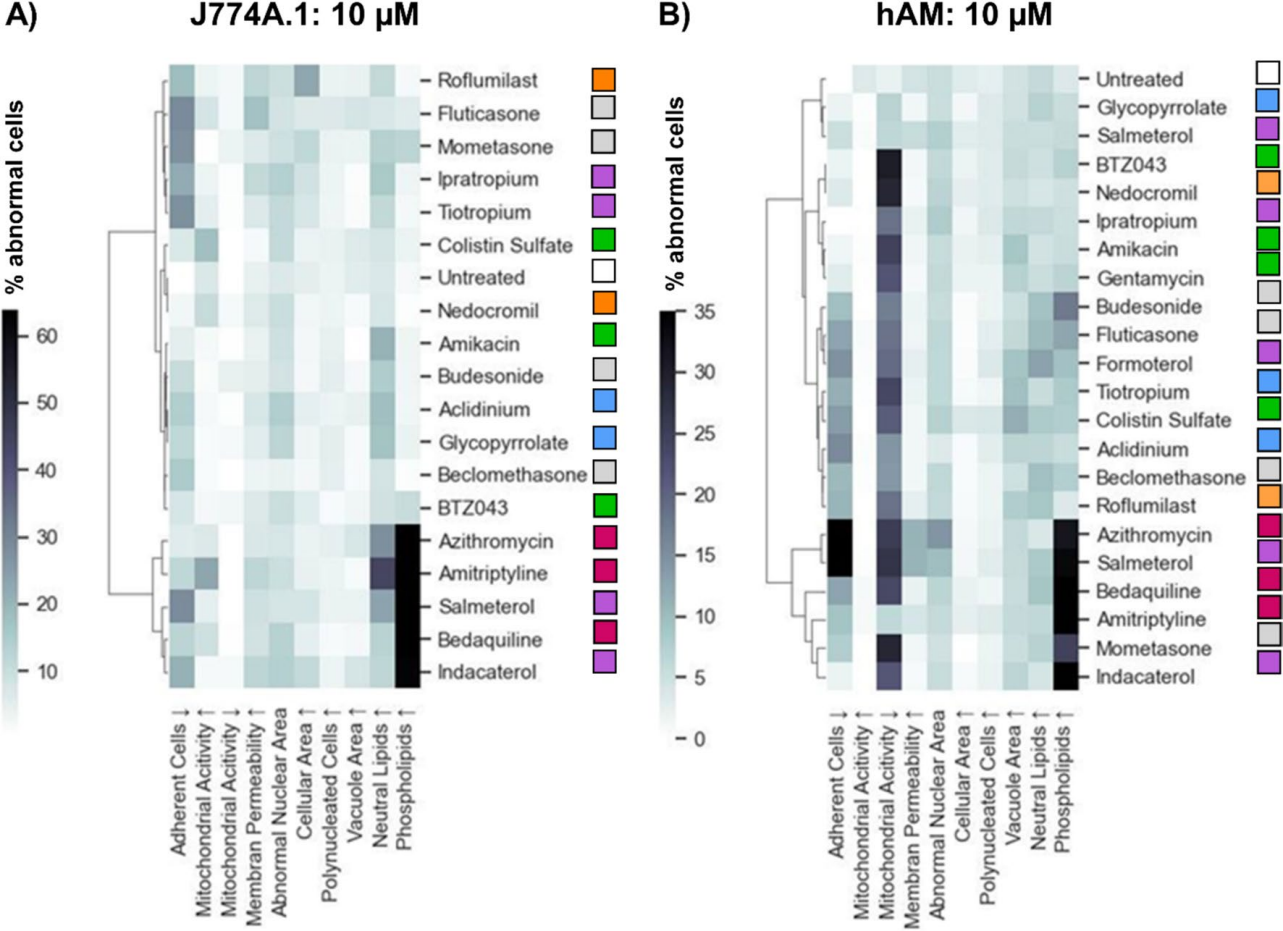
Hierarchical cluster map of (**A**) J774A.1 cells and (**B**) hAMs treated with selected compounds (10 µM). The heatmap values represent the intensity of the response. The color-coded dots next to each compound name indicate the pharmaceutical class (as defined in Table I).

Although the heatmaps shown above provide an efficient visual summary of the results, which is useful for the qualitative identification of response patterns in the data and comparison of cell types, they cannot provide insights into mathematical similarity between the data sets. To explore this, we employed hierarchical clustering analysis (Figs. 6 and 7). This method organises compounds into a tree-like structure (dendrogram) based on the similarity of their population response profiles. In our analysis, each compound was represented as a vector of features, including all measured responses (i.e., the nine HCIA parameters and the percentage of non-adherent cells). The similarity between these vectors was calculated using Euclidean distance. The clustering algorithm then iteratively grouped the most similar data points or clusters, revealing relationships between compounds based on their overall effect patterns. Clustering can be a useful tool when assessing novel compounds, as it may suggest potential similarities in cellular responses among different drugs and provide insights into toxicological behaviour prior to *in vivo* investigation, thereby aiding hypothesis-generation and guiding further investigations.

The hierarchical clustering analysis for J774A.1 cells treated with a 5 µM compound dose (Fig. 6A) very much reflects the response patterns visualised in the heatmaps (Figs. 3 and 4), whereby the strong treatment-related increases in phospholipid accumulation drive the similarity patterns. In this case, all CADs group together along with the beta-agonists indacaterol and salmeterol (recently classified as a CAD subgroup [25]). On the other end of the clustering spectrum, compounds with elevated neutral lipids have been grouped together. The pattern of clustering calculated for the hAM data (5 µM; Fig. 6B) show a very different picture. In this case, the inclusion of the data describing treatment-related loss of adherent cells (which was not depicted in the heatmaps), together with the phospholipid accumulation, drives the clustering on one end of the spectrum, while reductions in mitochondrial activity appear to drive clustering on the opposite end. Although the hierarchical clustering analysis roughly reflects trends observed in the hAM heatmaps (Fig. 5), it also reveals that hAM cells are more prone to detachment from the 96-well plates and therefore this is an important factor that should be included in the interpretation of toxicity.

The hierarchical clustering analysis for J774A.1 cells treated with a higher 10 µM compound dose (Fig. 7A) shows a high degree of similarity to the 5 µM clustering map, even though compounds with a higher cytotoxicity, such as amiodarone, had to be excluded from the analysis. Again, the CADs group together with the indacaterol and salmeterol. On the other end of the clustering spectrum, compounds with higher cell detachment have been grouped together. The pattern of clustering calculated for the hAM data (10 µM; Fig. 7B) is driven quite visually by phospholipid accumulation, reduction in mitochondrial activity and treatment-related loss of adherent cells.

## Discussion

The usefulness of HCIA as an experimental tool to both screen and better understand macrophage responses to drug treatment has been systematically explored in a series of investigations, whereby macrophage cells lines (rat NR8383, human U937 and THP-1, murine RAW264.7, J774A.1 and DMBM-2) have been compared with primary cell types (rat alveolar macrophage cells from lavage and human MDM with M1 and M2 phenotypes) [4, 14–16]. Furthermore, different incubation time points (typically 24 or 48 h), drug concentration ranges, pharmaceutical classes and image analysis methods (cell numbers evaluated, fluorescent microscopes, imaging software type and statistics), have been reported. The current study adds to this growing body of literature by contributing new data on the response patterns of murine J774A.1 macrophages to an extended panel of compounds across a wide dose range and compares these results to human alveolar macrophages obtained from bronchoscopy. The current study data was generated using a different fluorescence microscope and HCIA software compared to previous reports and demonstrates the comparability of the HCIA approach across various imaging platforms.

When considering the combined contributions of all studies published to date, it is possible to see a larger picture emerging. It is now apparent that cell lines, regardless of species of origin and compound incubation time, do not exhibit the same morphometric response patterns to drug treatment as primary alveolar macrophages obtained by lavage (Hoffman, 2017). However, within the groups of cell lines compared across studies, differentiated U937 cells appeared to be less responsive than the murine or rat-derived cell lines (Hoffman, 2017; Hoffmann, 2023). Some commonalities across cell types (cell lines and primary cells) can be observed, such as the reliable detection of phospholipid accumulation in all studies reporting this parameter. However, other parameters, such as elevated neutral lipids or impacts of drug treatment on nuclear area were highly variable across the studies. It follows that the use of convenient and inexpensive cell lines for large scale screening of drug candidates for responses such as DIPL can be performed to identify compounds which may have potential adverse effects, but such screening assays may not be suitable for understanding more complex mechanisms of drug responses. In this case, primary cells may be more suitable.

An evaluation of the more limited information on primary cell behaviour following CAD treatment showed a remarkable similarity in the HCIA response patterns between rat alveolar macrophages (Hoffman, 2017) and human alveolar macrophages investigated in this study. Since the procurement of alveolar macrophages by lavage is often impractical, expensive and, in the case of rat cells, contrary to efforts to reduce, refine or replace animal experiments, the proposal by Öhlinger *et al*. (2020) to use human blood-derived MDM cells for mechanistic studies of macrophage responses is an intriguing prospect [31]. Future studies investigating the response patterns of the MDMs with M1 and M2 phenotypes to a wider panel of pharmaceutical agents, such as described here and in Hoffman *et al*. (2023) would be useful to confirm whether MDM responses are similar to primary alveolar macrophages and whether the polarisation state influences response patterns to a larger extent than observed by Öhlinger *et al*. (2020) [4, 31].

Finally, two methods of HCIA data analysis were investigated in this study. The first method utilised the population quartile data to group responses into categories of: 1) non treatment-related, 2) possibly treatment-related, 3) likely treatment-related and 4) treatment-related. The response category was then plotted in a heat map to identify response patterns and compounds with treatment-related abnormalities. The second approach used the mean values of the percentage of abnormal cells in a hierarchical clustering analysis to group compounds with similar response patterns. Both methods reflected the same trends in the data sets. The first method, however, was a more suitable tool for rapid “go-no go” decision-making, as it provided a simple and easy categorisation of the response to treatment. The hierarchical clustering analysis, in contrast, is perhaps less easy to use to establish if a response is treatment-related but can be useful for identifying prevalent mechanisms of cell responses and showing which compounds produce similar responses.

Future research should focus not only on expanding the panel of fluorescent markers (including additional relevant biomarkers) but also on offering deeper insights into macrophage biology, such as the impact of the polarisation state on the cellular responses to drug compounds. Furthermore, investigating the usefulness of HCIA in screening other cell types, such as epithelial cells, could broaden its utility in the drug development process. Additionally, the translation of these *in vitro* results to *in vivo* effects still requires careful consideration and validation.

## Conclusions

This study has contributed new and useful information characterising multi-parameter HCIA for the early detection of alveolar macrophage responses to a wide panel of pharmaceutical compounds. Crucially, we demonstrated that human primary alveolar macrophages responded differently to drug treatment than macrophage cell lines. Comparison with literature data show that the human primary alveolar macrophages show more similar response patterns to primary rat alveolar macrophages, while cell lines (regardless of the species of origin) show different response profiles with a high degree of similarity to each other. Therefore, while the integration of multiple parameters provides a more comprehensive assessment of cellular responses to drug candidates, interpretation of HCIA data sets should be made with these differences in mind. At the same time, it is important to recognise that the detection of phospholipid accumulation within cells (as a single parameter for the screening of DIPL) was highly robust across all cell types tested, not only in this study but in several others. This observation suggests that a variety of macrophage-like cell types (regardless of species of origin, primary or cell line) can be used for DIPL screening of novel compounds. On the other hand, the choice of cell type may be more crucial for mechanistic studies on macrophage biology and further research characterising the response patterns of MDM with different phenotypes to a wider panel of pharmaceutical agents would be an important next step.

## Supplementary Information

Below is the link to the electronic supplementary material.

Supplementary file1 (XLSX 102 KB)

Supplementary file2 (XLSX 216 KB)

Supplementary file3 (DOCX 987 KB)

## Data Availability

The datasets generated during and/or analysed during the current study are available in the [NAME] repository, [PERSISTENT WEB LINK TO DATASETS]”.
