# Supplementary material for: High Content Image Analysis of Cellular Responses of the Murine J774A.1 Cell Line and Primary Human Cells Alveolar Macrophages to an Extended Panel of Pharmaceutical Agents
Source: Pharm Res. 2025 Jan 7;42(1):93–108. doi: 10.1007/s11095-024-03806-y (PMC11785622; doi:10.1007/s11095-024-03806-y)
Supplement: Supplementary file 3 — Supplementary file3 (DOCX 987 KB) [file 11095_2024_3806_MOESM3_ESM.docx]

**Supplementary Information**

**High content image analysis of cellular responses of the murine J774A.1 cell line and primary human cells alveolar macrophages to an extended panel of pharmaceutical agents**

Lysann Tietze^1^, Laura Urbano^3^, Stephan Eisenmann ^2^, Jacqueline Schwarzinger^5,6^, Julia Kollan^1^, Ben Forbes^4^, Lea Ann Dailey^1,5^, Gabriela Hädrich^1,5^

^1^Department of Visceral, Transplant, Thoracic and Vascular Surgery, University of Leipzig Medical Center, 04103 Leipzig, Germany.

^2^ Department of Pulmonary Medicine, University Hospital of Halle-Wittenberg, Halle (Saale) 06120, Germany

^3^ Department of Clinical, Pharmaceutical and Biological Sciences, University of Hertfordshire, College Lane, Hatfield, Hertfordshire AL10 9AB, U.K.

^4^ Institute of Pharmaceutical Science, Faculty of Life Sciences and Medicine, King’s College London, London [SE1 9NH](https://www.bing.com/local?lid=YN1029x102583675&id=YN1029x102583675&q=Faculty+Of+Pharmaceutical+Medicine&name=Faculty+Of+Pharmaceutical+Medicine&cp=51.5251350402832%7e-0.14546699821949005&ppois=51.5251350402832_-0.14546699821949005_Faculty+Of+Pharmaceutical+Medicine), UK

^5^ Department of Pharmaceutical Sciences, University of Vienna, Vienna 1090, Austria.

^6^Vienna Doctoral School of Pharmaceutical, Nutritional and Sport Sciences, University of Vienna, 1090 Vienna, Austria

Corresponding author

Prof. Dr. Lea Ann Dailey

[leaann.dailey@univie.ac.at](mailto:leaann.dailey@univie.ac.at),

[+43-1-4277-55499](https://uphone.univie.ac.at/tweb/portal/req/ClickToDial?clickToDial=+431427755499)

**Table S1.** Mean, standard deviation and RSD% of the percentage of abnormal J774A.1 cells following treatment with a low and high dose of six selected compounds for all 10 parameters. The values are derived from n=6 experiments with different passage numbers. Values for untreated J774A.1 cells (n=20) are depicted in grey for comparison.

| Non-adherent cells | Low dose | | | High dose | | |
| --- | --- | --- | --- | --- | --- | --- |
|  | Mean | SD | RSD% | Mean | SD | RSD% |
| Amiodarone | 4.0 | 5.8 | 145 | 17.7 | 19.0 | 108 |
| Budesonide | 3.3 | 8.2 | 245 | 11.3 | 15.5 | 136 |
| Campothecin | 4.5 | 10.5 | 234 | 24.7 | 12.9 | 52 |
| Tiotropium | 19.8 | 18.0 | 91 | 16.7 | 24.6 | 147 |
| Indacaterol | 9.0 | 14.2 | 158 | 13.8 | 17.9 | 130 |
| Amikacin | 4.8 | 10.9 | 225 | 5.0 | 12.2 | 245 |
| Untreated cells* | - | - | - |  |  |  |

**The percentage non-adherent cells for the untreated sample group is defined as 0%.*

| Mitochondrial Activity ⇧ | Low dose | | | High dose | | |
| --- | --- | --- | --- | --- | --- | --- |
|  | Mean | SD | RSD% | Mean | SD | RSD% |
| Amiodarone | 4.8 | 4.1 | 86 | 23.0 | 10.3 | 45 |
| Budesonide | 1.9 | 1.2 | 63 | 1.3 | 1.3 | 108 |
| Campothecin | 1.1 | 0.6 | 52 | 0.8 | 1.2 | 150 |
| Tiotropium | 3.3 | 3.9 | 117 | 4.8 | 3.8 | 80 |
| Indacaterol | 4.4 | 2.9 | 67 | 3.3 | 3.1 | 95 |
| Amikacin | 2.7 | 2.4 | 88 | 2.8 | 3.3 | 120 |
| Untreated cells | 5.9 | 1.7 | 30 |  |  |  |

| Mitochondrial Activity ⇩ | Low dose | | | High dose | | |
| --- | --- | --- | --- | --- | --- | --- |
|  | Mean | SD | RSD% | Mean | SD | RSD% |
| Amiodarone | 0.2 | 0.3 | 155 | 0.2 | 0.2 | 138 |
| Budesonide | 1.0 | 1.1 | 107 | 1.9 | 2.6 | 134 |
| Campothecin | 5.1 | 3.7 | 72 | 15.0 | 17.2 | 114 |
| Tiotropium | 0.9 | 1.7 | 200 | 0.7 | 1.2 | 167 |
| Indacaterol | 0.9 | 1.6 | 189 | 1.7 | 3.8 | 223 |
| Amikacin | 0.4 | 0.5 | 136 | 1.3 | 2.2 | 170 |
| Untreated cells | 0.5 | 0.2 | 43 |  |  |  |

| Membrane Permeability ⇧ | Low dose | | | High dose | | |
| --- | --- | --- | --- | --- | --- | --- |
|  | Mean | SD | RSD% | Mean | SD | RSD% |
| Amiodarone | 4.8 | 2.2 | 46 | 11.8 | 3.6 | 30 |
| Budesonide | 2.4 | 1.7 | 70 | 3.8 | 2.7 | 71 |
| Campothecin | 2.7 | 2.2 | 80 | 3.7 | 2.3 | 65 |
| Tiotropium | 12.3 | 10.2 | 82 | 6.7 | 3.1 | 46 |
| Indacaterol | 5.0 | 2.5 | 51 | 7.0 | 4.3 | 61 |
| Amikacin | 3.7 | 1.7 | 46 | 3.4 | 1.3 | 39 |
| Untreated cells | 4.8 | 0.3 | 7 |  |  |  |

| Abnormal Nuclear Area | Low dose | | | High dose | | |
| --- | --- | --- | --- | --- | --- | --- |
|  | Mean | SD | RSD% | Mean | SD | RSD% |
| Amiodarone | 5.8 | 3.2 | 55 | 7.4 | 1.8 | 24 |
| Budesonide | 1.9 | 0.8 | 41 | 2.6 | 1.4 | 52 |
| Campothecin | 6.6 | 5.6 | 84 | 18.4 | 7.1 | 39 |
| Tiotropium | 7.4 | 3.8 | 51 | 4.5 | 3.5 | 78 |
| Indacaterol | 9.4 | 2.7 | 29 | 9.9 | 2.5 | 25 |
| Amikacin | 5.0 | 1.2 | 25 | 6.1 | 2.7 | 44 |
| Untreated cells | 6.5 | 1.0 | 15 |  |  |  |

| Cellular Area ⇧ | Low dose | | | High dose | | |
| --- | --- | --- | --- | --- | --- | --- |
|  | Mean | SD | RSD% | Mean | SD | RSD% |
| Amiodarone | 3.8 | 1.8 | 48 | 15.4 | 7.2 | 46 |
| Budesonide | 1.1 | 0.6 | 54 | 1.9 | 0.9 | 46 |
| Campothecin | 1.9 | 1.5 | 80 | 9.4 | 4.7 | 51 |
| Tiotropium | 6.1 | 2.8 | 46 | 6.4 | 4.9 | 77 |
| Indacaterol | 7.5 | 2.2 | 29 | 7.3 | 2.7 | 37 |
| Amikacin | 3.0 | 1.5 | 50 | 1.9 | 0.6 | 32 |
| Untreated cells | 5.5 | 0.5 | 8 |  |  |  |

| Polynucleated Cells ⇧ | Low dose | | | High dose | | |
| --- | --- | --- | --- | --- | --- | --- |
|  | Mean | SD | RSD% | Mean | SD | RSD% |
| Amiodarone | 4.0 | 1.5 | 38 | 5.2 | 2.1 | 40 |
| Budesonide | 6.2 | 3.2 | 53 | 4.4 | 2.2 | 49 |
| Campothecin | 3.2 | 0.9 | 30 | 2.5 | 0.9 | 34 |
| Tiotropium | 4.5 | 3.5 | 78 | 4.5 | 2.9 | 65 |
| Indacaterol | 4.4 | 3.3 | 74 | 4.2 | 2.5 | 61 |
| Amikacin | 4.6 | 3.1 | 68 | 5.3 | 2.3 | 44 |
| Untreated cells | 3.4 | 0.4 | 12 |  |  |  |

| Vacoule Area ⇧ | Low dose | | | High dose | | |
| --- | --- | --- | --- | --- | --- | --- |
|  | Mean | SD | RSD% | Mean | SD | RSD% |
| Amiodarone | 13.2 | 21.1 | 160 | 20.0 | 16.9 | 85 |
| Budesonide | 6.5 | 9.2 | 142 | 3.5 | 5.2 | 152 |
| Campothecin | 1.3 | 2.3 | 169 | 7.7 | 10.4 | 136 |
| Tiotropium | 5.4 | 6.9 | 128 | 7.8 | 13.9 | 178 |
| Indacaterol | 3.5 | 4.4 | 125 | 5.2 | 2.7 | 53 |
| Amikacin | 6.1 | 7.1 | 116 | 2.2 | 2.3 | 105 |
| Untreated cells | 5.4 | 1.0 | 17 |  |  |  |

| Neutral Lipids ⇧ | Low dose | | | High dose | | |
| --- | --- | --- | --- | --- | --- | --- |
|  | Mean | SD | RSD% | Mean | SD | RSD% |
| Amiodarone | 8.6 | 3.0 | 35 | 24.9 | 13.6 | 55 |
| Budesonide | 12.2 | 12.6 | 103 | 25.3 | 20.0 | 83 |
| Campothecin | 6.7 | 5.3 | 79 | 15.8 | 12.9 | 81 |
| Tiotropium | 11.3 | 14.4 | 128 | 13.1 | 14.2 | 109 |
| Indacaterol | 4.2 | 2.8 | 67 | 19.3 | 22.2 | 115 |
| Amikacin | 9.9 | 6.1 | 61 | 23.4 | 20.4 | 87 |
| Untreated cells | 5.4 | 1.0 | 19 |  |  |  |

| Phospholipids ⇧ | Low dose | | | High dose | | |
| --- | --- | --- | --- | --- | --- | --- |
|  | Mean | SD | RSD% | Mean | SD | RSD% |
| Amiodarone | 3.5 | 1.1 | 32 | 99.6 | 0.7 | 1 |
| Budesonide | 2.7 | 1.2 | 45 | 7.1 | 6.5 | 92 |
| Campothecin | 2.1 | 2.0 | 97 | 1.6 | 1.6 | 102 |
| Tiotropium | 5.9 | 3.9 | 65 | 5.1 | 1.7 | 34 |
| Indacaterol | 5.3 | 2.5 | 47 | 28.7 | 15.0 | 52 |
| Amikacin | 5.7 | 2.3 | 41 | 5.7 | 2.9 | 51 |
| Untreated cells | 1.4 | 1.0 | 66 |  |  |  |

**Comparing abnormal nuclear area and elevated neutral lipid response patterns in two representative pharmaceutical compound classes: Beta-agonist and anticholinergics**

The heatmaps in Figures 3-4 of the main manuscript indicate that compound treatment in J774 cells frequently results in abnormal nuclear area and elevated neutral lipids, although the exact mechanism behind these responses is not fully clarified by the HCIA itself. To interrogate whether the phys-chem properties, especially lipophilicity, of compounds with similar structures influence the percentage of cells with abnormal nuclear area or neutral lipid content, two compound classes, beta-agonists (**Figure S1**) and anticholinergics (**Figure S2**), were selected and the data distribution for these two endpoints was plotted at each applied dose. The colouring of the box and whisker plots indicates the category assigned to the response: white = not treatment relation, light shading = possibly treatment related, dark shading = likely treatment related.


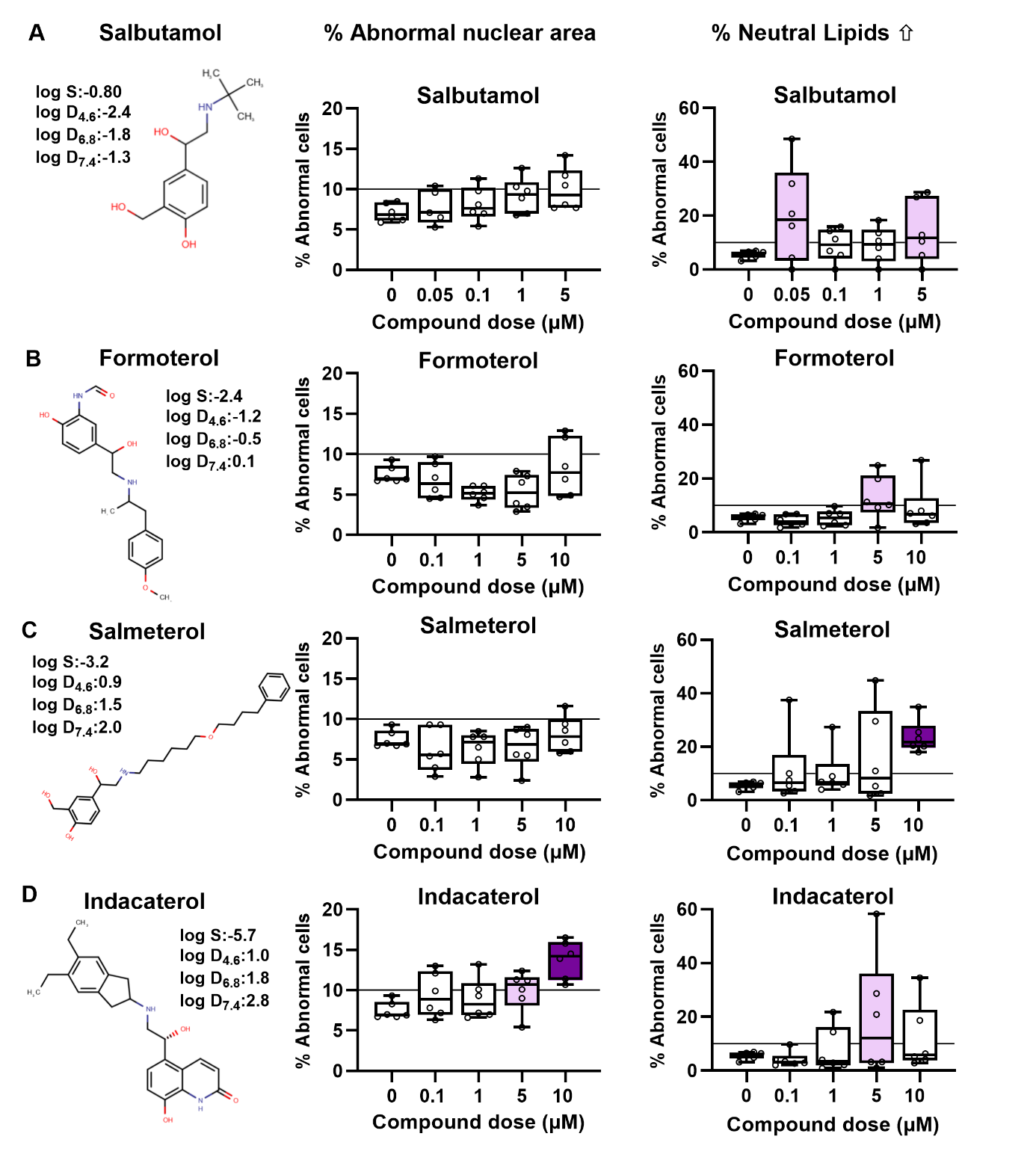


**Figure S1.** Data distribution (box and whisker plots) of the percentage of J774A.1 cells with abnormal nuclear area and elevated neutral lipid content following 48 h incubation with increasing doses of A) salbutamol, B) formoterol, C) salmeterol and D) indacaterol. Box limits depict the range of the central 50% of the data, with a line marking the median value. Whiskers show the minimum and maximum values from n=6 experiments with different passage numbers. Structural information is provided for each compound and the predicted log S (solubility; mg/mL) and log D values were calculated using Chemicalize (30.11.2024, <http://chemicalize.com/> , developed by ChemAxon.)

In both compound groups, no discernible trends relating pH-dependent compound lipophilicity to percentage of cells with abnormalities were observed. It is also clear to see that the categorization of the response depends not only on the percentage of abnormal cells in the population but also on the data distribution. For example, it can be observed that replicate values of cell populations with abnormal nuclear area are more narrowly distributed, i.e. reproducible, than cell populations with elevated neutral lipid staining. Within the two data sets shown, it appears that some cell passage numbers show greater variations in the percentage of cells with elevated content lipid content than other passage numbers. To provide further clarity and transparency, all replicate values for each parameter are provided for the J774 and hAM cells (see provided Excel spreadsheets in the supplementary information).


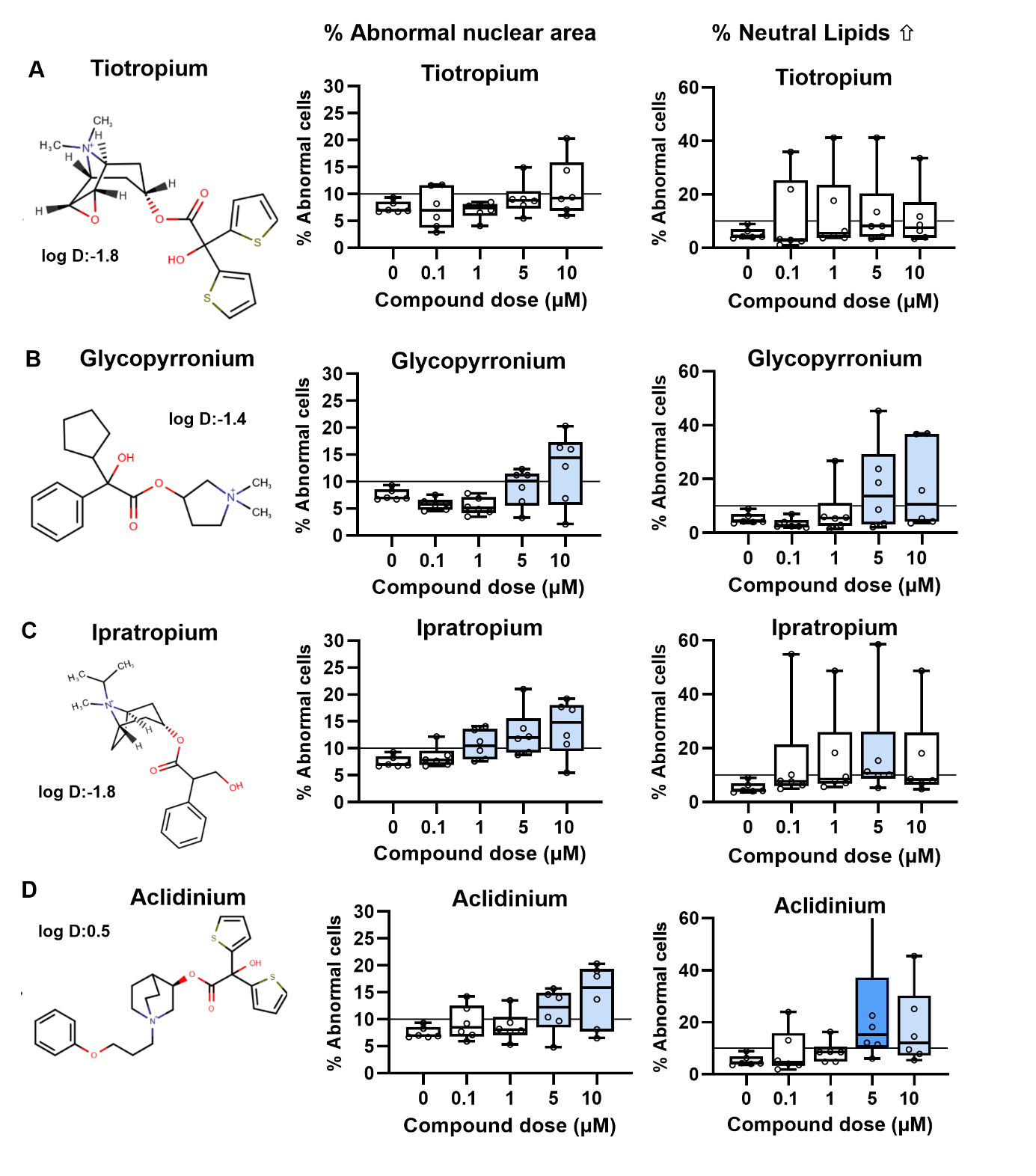


**Figure S2.** Data distribution (box and whisker plots) of the percentage of J774A.1 cells with abnormal nuclear area and elevated neutral lipid content following 48 h incubation with increasing doses of A) tiotropium, B) glycopyrronium, C) iprotropium, and D) aclidinium. Box limits depict the range of the central 50% of the data, with a line marking the median value. Whiskers show the minimum and maximum values from n=6 experiments with different passage numbers. Structural information is provided for each compound and the predicted log S (solubility; mg/mL) and log D values were calculated using Chemicalize (30.11.2024, <http://chemicalize.com/> , developed by ChemAxon.) pK_a_ prediction cannot be performed since the molecules contain no readily ionizable atom forms.
